# New insights into phylogenetic relationships of Rhabdocoela (Platyhelminthes) including members of Mariplanellida

**DOI:** 10.1186/s40850-023-00171-y

**Published:** 2023-07-11

**Authors:** Íñigo Vicente-Hernández, Werner Armonies, Katharina Henze, M. Teresa Aguado

**Affiliations:** 1 Animal Evolution & Biodiversity, Georg-August-Universität Göttingen, 37073 Göttingen, Germany; 2 Alfred Wegener Institute Helmholtz Centre for Polar and Marine Research, Wattenmeerstation Sylt, Hafenstr. 43, 25992 List, Germany

**Keywords:** Flatworms, Phylogeny, Free-living Platyhelminthes, Maximum likelihood, Bayesian Inference, 18S, 28S, Kalyptorhynchia

## Abstract

**Background:**

Previous flatworm phylogenetic research has been carried out analysing 18S and 28S DNA markers. Through this methodology, Mariplanellinae subfamily has been recently re-classified as Mariplanellida status novus. This new classification implied that 3 genera belonged to Mariplanellida: *Mariplanella*, *Lonchoplanella* and *Poseidoplanella*. In this study, we aim to clarify some of the relationships within Rhabdocoela analysing 18S and 28S DNA markers of a total of 91 species through Maximum Likelihood and Bayesian Inference methodologies. A total of 11 species and genera, including *Lonchoplanella*, from the island of Sylt are included and had not previously been involved in any molecular phylogenetic analyses.

**Results:**

Our phylogenetic results support Mariplanellida as an independent group within Rhabdocoela and its status as an infraorder. Our study suggests that *Lonchoplanella axi* belongs to Mariplanellida. Within Rhabdocoela, *Haloplanella longatuba* is nested within Thalassotyphloplanida, instead of Limnotyphloplanida. Within Kalyptorhynchia, the taxon Eukalyptorhynchia turned out to be paraphyletic including members of Schizorhynchia. These results also support the position of the genus *Toia* separate from Cicerinidae*.*

**Conclusions:**

*Lonchoplanella axi* belongs to Mariplanellida, whose status as infraorder is herein confirmed. The genus *Toia* belongs separate from Cicerinidae. Further research is needed to clarify the phylogenetic relationships of *Hoploplanella*. Most of the species, genera and families included in this study with more than one terminal are monophyletic and well supported. Adding gene markers and complementary morphological studies will help to clarify those relationships that remain uncertain.

**Supplementary Information:**

The online version contains supplementary material available at 10.1186/s40850-023-00171-y.

## Background

Flatworms are a large group in terms of diversity, with more than 26,500 described species [1, 2]. Most of them are parasitic, while around 6500 species of them are “free-living Platyhelminthes”. The parasitic flatworms, Neodermata (comprising Trematoda, Monogenea, and Cestoda), are a well-defined and supported clade characterized by a syncytial, nonciliated epidermis whose nuclei-bearing parts lie sunken below the musculature. The rest of platyhelminthes are mostly free-living but also symbiotic flatworms, also known as turbellarians (non-cladistic group); most of them with a ciliated, cellular epidermis [3].

The free living Rhabdocoela and Proseriata are the two most diverse microturbellarian orders, which, in recent phylogenetic hypotheses, are basally branching within Euneoophora [4] (Sup. Figure 1). Proseriata has above 400 described species [5], and Rhabdocoela, has about 1530 described species [6]. Nevertheless, these numbers represent a scarce amount of the estimated total number of microturbellarian species present on Earth, which was estimated to be around 44.000 by Armonies [7]. Further research is needed in order to record their diversity more accurately [8].

**Fig. 1:**
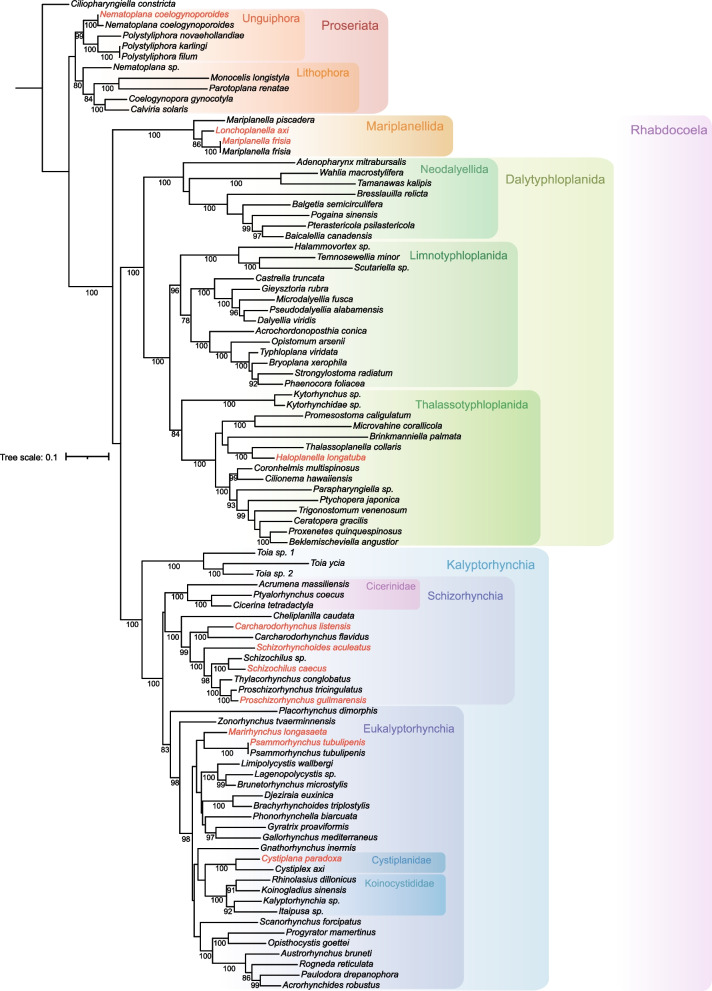
ML phylogenetic tree inferred from the concatenated data set (18S+28S). Species provided by this study in red. Bootstrap support values under/beside nodes. Values below 70% not represented

Proseriata, is a monophyletic order [9]; based on morphological traits Sopott-Ehlers [10] divided it into Unguiphora (taxa without cirrus and a statocyst but with pigment in the mantle cells of rhabdomeric receptors) and Lithophora (statocyst present and no pigment in the mantle cells). Later the monophyly of both clades was confirmed [9, 11]), though with some dispute in the Unguiphora as it was underrepresented in the studies supporting its monophyly and appears to be paraphyletic in others [11, 12]). Within Proseriata, the position of the genus *Ciliopharyngiella* Ax, 1952 has been recently debated. Curini-Galletti et al. [11] suggested that it belongs inside Proseriata clustered with Unguiphora, while Van Steenkiste & Leander [13] suggested *Ciliopharyngiella* being the sister lineage of Proseriata.

Rhabdocoela is a monophyletic order [9] of free-living Platyhelminthes, traditionally subdivided into two major groups: Kalyptorhynchia and Dalytyphloplanida, with and without an anterior proboscis, respectively. Within Rhabdocoela, the family Mariplanellidae was initially included in Dalytyphloplanida though not fully fitting with the diagnostic characters. Recently, Steenkiste & Leander [13] re-classified the subfamily Mariplanellinae to Mariplanellida status novus, representing the monophyletic group sister to a large clade comprising Kalyptorhynchia and Dalytyphloplanida. According to this classification, Mariplanellidae (the only family of Mariplanellida) currently comprises three genera: *Mariplanella* Ax & Heller, 1970, *Lonchoplanella* Ehlers, 1974, and *Poseidoplanella* Willems et al., 2005. Morphologically, these genera share the characters single ovary and a double connection in the female reproductive system. However, the phylogenetic analyses by Van Steenkiste and Leander [13] only included two species of *Mariplanella, M. piscadera* Van Steenkiste & Leander, 2022 and *M. frisia* Ax & Heller, 1970, while members of *Lonchoplanella* and *Poseidoplanella* have not been included in any molecular phylogenetic analysis up to date.

## Results

Trees obtained from independent Maximum Likelihood (ML) analyses of single markers (18S, 28S), as well as the one obtained from the analyses of the concatenated data matrix (18S + 28S) showed congruent results (SFig. 2, SFig. 3 and Fig. 1, respectively). The concatenated data matrix (18S + 28S) run through Bayesian Inference (BI) yielded highly congruent results (SFig. 4) with practically the same tree topology and high support values. The trees were rooted in *Ciliopharyngiella constricta* Martens & Schockaert, 1981, excepting the tree of 28S which was rooted in Proseriata.

In the ML concatenated analysis (Fig. 1), the taxon Proseriata is not well supported (68 B, where B represents bootstrap support values). Within Proseriata, Unguiphora (excepting *Nematoplana* sp.) and Lithophora are monophyletic and well supported (99B and 80B, respectively). Within Unguiphora, both *Nematoplana coelogynoporoides* Meixner, 1938 are in a monophyletic group; however, *Nematoplana* sp. is sister to Lithophora. The genus *Polystyphora* Ax, 1958, represented by three species, is monophyletic. Lithophora is subdivided into Otoplanidae and Monocelididae (100 B), and Coelogynoporidae and Calviriidae (100 B), respectively, as sister groups.

Rhabdocoela is well supported (100 B). Within Rhabdocoela, the three clades, Mariplanellida, Dalytyphloplanida and Kalyptorhynchia, are strongly supported (100 B). Mariplanellida is sister to Dalytyphloplanida and Kalyptorhynchia, which are joined in a not highly supported clade (69 B). In the concatenated analysis (Fig. 1), the species *Lonchoplanella axi* is located within Mariplanellida, sister to *M. axi* (86 B), showing the genus *Mariplanella*, as currently delineated, as paraphyletic. The 18S tree (SFig. 2) shows *Mariplanella* as monophyletic but is not supported (61 B) with *Lonchoplanella axi* as its sister group. Within Dalytyphloplanida, Neodalyellida, Limnotyphloplanida (excepting *Haloplanella longatuba* Ax & Heller, 1970*)* and Thalassotyphloplanida are monophyletic and, excepting the latter, well supported (100 B, 100 B, 96 B, and 84 B, respectively). Limnotyphloplanida and Thalassotyphloplanida are sister to each other (100B). The species *Haloplanella longatuba* is located within Thalassotyphloplanida, out of the Limnotyphloplanida.

Within Kalyptorhynchia, Eukalyptorhynchia is paraphyletic, including Schizorhynchia. The genus *Toia* Markus, 1952 is located as the sister group of a large clade (100 B) containing the rest of Eukalyptorhynchia (83 B) and Schizorhynchia, which is not supported (48B). These two latter groups are better supported in the BI analisis (SFig. 4) with a posterior probability value of 1. The family Cheliplanidae appears to be paraphyletic (100 B) with *Cheliplanilla caudata* Meixner 1938 being the sister taxon of a group compromised by *Carcharodorhynchus* Meixner, 1938 and Schizorhynchidae, represented by more than one species, and also well supported (100 B). The genera *Carchadorhynchus*, *Schizochilus* Boaden, 1963 and *Proschizorhynchus* Meixner, 1928, and *Psammorhynchus tubulipenis* Meixner, 1938, including more than one terminal, are respectively monophyletic, though herein represented by few species.

## Discussion

Our results show Mariplanellida as an independent group within Rhabdocoela as found by Van Steenkiste & Leander [13], and previously suggested by other molecular analyses [9]. Its higher-level status as an additional infraorder proposed by Van Steenkiste & Leander [13] within Rhabdocoela is supported herein as well. Our study included *Lonchoplanella axi*, a genus and species not incorporated before in a molecular phylogeny. *Lonchoplanella axi* shares a number of conspicuous characters with *Mariplanella frisia*, including two types of adenal rhabdites (needle-shaped and elongate viscous) and a muscular copulatory bursa with a sclerotized basal membrane [13] and had been described as a member of Mariplanellidae [14]. The topology of the concatenated tree confirms *Lonchoplanella axi* as belonging to the Mariplanellidae family and Mariplanellida infraorder. More information (terminals and markers) is needed to clarify the monophyly of both genera, and further analyses including *Poseidoplanella halleti* Willems et al., 2005 (presumably the only missing taxon of Mariplanellidae) will clarify the relationships between the three genera.

Our study included six new terminals and five species of Kalyptorhynchia in addition to those included by Van Steenkiste & Leander [13]. Results corroborate Eukalyptorhynchia as non-monophyletic supporting Willems et al. [9] and Tessens et al. [15] results. Our results also support the position of the genus *Toia* separate from Cicerinidae, as suggested by Tessens, et al. [15]. The rest of Cicerinidae are monophyletic and the sister group of Schizorhynchia. Cicerinidae (except *Toia)* were located in a polytomy with Schizorhynchia and most members of Eukalyptorhynchia in the analysis of Van Steenkiste & Leander [13].

Most of the species, genera and families included in this study with more than one terminal are monophyletic and well supported. However, the terminals *Nematoplana* sp. and *Haloplanella longatuba* need further consideration. Within Proseriata, *Nematoplana* sp. (downloaded from GenBank) is not closely related to *N. coelogynoporoides;* its sister relationship to Lithophora might indicate that this terminal could have been misidentified or the result of contamination in the 18S sequence. Additionally, the length of branches of *Nematoplana coelogynoporoides* from Sylt (included herein) and *N. coelogynoporoides* from Roscoff (downloaded from GenBank) might indicate they are not be the same species.

In Rhabdocoela, *Haloplanella longatuba* is one of the species we incorporate in a phylogenetic analysis for the first time with molecular information. The relationships of brackish and marine water Typhloplanidae species, such as *Haloplanella longatuba* have been previously discussed [6, 16, 17]. Rieger [16] describes the resemblance between certain genera in the family Typhloplanidae (Limnotyphloplanida parvorder) and various genera in the Thalassotyphloplanida parvorder, thus encouraging future reorganizations within these taxonomic groups. Hochberg & Cannon [17] remarks on the presence of an unusual character in some genera of the Typhloplanidae family, such as *Haloplanella* Luther, 1946 and *Pratoplana* Ax 1960, where a stylet is present in the copulatory apparatus instead of a cirrus, which is one of the family's ground pattern traits. Moreover, several similarities between this species and members of Thalassotyphloplanida have been found, such as the female genital canal and the proboscis structure. Van Steenkiste et al. [6] already suggested that several brackish water and marine Typhloplanidae taxa might be closely related to Byrsophlebidae (Thalassotyphloplanida). Our results show *Haloplanella longatuba* nested within Thalassotyphloplanida, which supports that the position of this taxon has to be taken into further consideration, and revised in future studies.

In this study, the selected gene markers, 18S and 28S were used because they are already available for a large number of species and have been previously found useful to discern phylogenetic relationships within Proseriata and Rhabdocoela [13]. Nevertheless, Next Generation Sequencing (NGS) techniques will be undoubtedly useful to discern and clarify the evolution of these groups.

## Conclusions

Our results support the infraorder Mariplanellida as an independent group within Rhabdocoela and confirms, by the first time with molecular data, that *Lonchoplanella axi* belongs to Mariplanellida. Eukalyptorhynchia is paraphyletic including members of Schizorhynchia, and the genus *Toia* separate from Cicerinidae. *Haloplanella longatuba* is herein nested within Thalassotyphloplanida, and not in Typhloplanidae (Limnotyphloplanida), which suggests that further studies are needed to clarify its phylogenetic relationships.

More terminals and information from morphological studies, as well as new markers of NGS techniques may clarify the gaps and the still doubtful relationships.

## Material and methods

In this study we aim to provide a more robust phylogenetic hypothesis of Rhabdocoela relationships. For this purpose, we introduced the species *Lonchoplanella axi* to phylogenetic analyses to test whether or not it belongs to *Mariplanellida*. With respect to Kalyptorhynchia and Dalytyphloplanida we added species of *Cystiplana* Karling, 1964*, Haloplanella, Marirhynchus* Schilke, 1970*, Lonchoplanella,* and *Schizorhynchoides* Meixner, 1928 (Table 1).

**Table 1:** Terminals included, genes and GenBank Accession numbers. Species provided by this study are represented in bold

	18S	28S	Location
PROSERIATA			
*Ciliopharyngiella constricta*	AY775754	–	Belgium: Oostende, Mariakerke
*Nematoplana coelogynoporoides*	KJ682383	KJ682445	France: Roscoff
*Nematoplana* sp.	AJ270160	AJ270175	Australia:Shelly River, Queensland
***Nematoplana coelogynoporoides***	**OP604379**	**––-**	**Germany: Sylt**
*Polystyliphora novaehollandiae*	AJ270161	AJ270177	Unknown
*Parotoplana renatae*	AJ012517	AJ270176	Unknown
*Calviria solaris*	AJ270153	AJ270168	Unknown
*Coelogynopora gynocotyla*	AJ243679	AJ270170	Unknown
*Monocelis longistyla*	KR364618	KR364663	Italy: La Maddalena, Sardinia
RHABDOCOELA			
Mariplanellida *status novus*			
*Mariplanella frisia*	AJ012514	–	Germany: Sylt
***Mariplanella frisia***	**OP604380**	**OP604370**	**Germany: Sylt**
***Lonchoplanella axi***	**OP604381**	**OP604369**	**Germany: Sylt**
*Mariplanella piscadera* sp. nov	OM339545	OM339542	Curacao
Kalyptorhynchia			
*Toia* sp. 1	OM339546	OM339543	Canada
*Toia* sp. 2	OM339547	OM339544	Canada
*Toia ycia*	KC869828	KC869881	Unknown
*Cheliplanilla caudata*	KJ887449	KJ887502	Sweden: Tjörn
***Carcharodorhynchus listensis***	**OP604377**	**OP604363**	**Germany: Sylt**
*Carcharodorhynchus flavidus*	KJ887457	KJ887563	Spain: Lanzarote, Orzola
***Cystiplana paradoxa***	**––**	**OP604368**	**Germany: Sylt**
***Proschizorhynchus gullmarensis***	**OP604375**	**OP604364**	**Germany: Sylt**
*Proschizorhynchus tricingulatus*	KJ887423	KJ887503	Spain: Lanzarote, Caleton Blanco
***Marirhynchus longasaeta***	**OP604374**	**OP604367**	**Germany: Sylt**
***Schizochilus caecus***	**OP604376**	**OP604365**	**Germany: Sylt**
*Schizochilus sp.*	KR339044	KR339059	USA: Emerald Isle Site
***Schizorhynchoides aculeatus***	**OP604378**	**OP604366**	**Germany: Sylt**
*Thylacorhynchus conglobatus*	KJ887448	KJ887534	Germany: Sylt
*Acrumena massiliensis*	KJ887417	KJ887509	Italy: Sardinia
*Cicerina tetradactyla*	KJ887465	KJ887520	Sweden: Sandhammar
*Ptyalorhynchus coecus*	KJ887416	KJ887550	Belgium: Ostend
*Placorhynchus dimorphis*	KJ887409	KJ887507	Finland: Tvärminne
*Zonorhynchus tvaerminnensis*	KJ887455	KJ887516	Finland: Henriksberg
*Cystiplex axi*	KJ887437	KJ887549	Italy: Sardinia
*Koinogladius sinensis*	MF443159	MF443174	China
*Rhinolasius dillonicus*	MW081602	MW054461	Unknown
***Psammorhynchus tubulipenis***	**OP604373**	**––**	**Germany: Sylt**
*Psammorhynchus tubulipenis*	KJ887438	KJ887561	Germany: Sylt
*Gnathorhynchus inermis*	KJ887402	KJ887524	Germany: Sylt
*Brachyrhynchoides triplostylis*	KJ887399	KJ887558	Italy: Sardinia
*Djeziraia euxinica*	KJ887442	KJ887527	Italy: Sardinia
*Limipolycystis wallbergi*	KJ887467	KJ887491	Italy: Sardinia
*Brunetorhynchus microstylis*	KJ887468	KJ887494	France: Banyuls-sur-mer
*Lagenopolycystis mandelai*	KJ887441	KJ887536	South Africa: iSimangaliso NP
*Phonorhynchella biarcuata*	KJ887447	KJ887548	Sweden: Kattegat
*Gallorhynchus mediterraneus*	KJ887428	KJ887496	Italy: Sardinia
*Gyratrix proaviformis*	KJ887430	KJ887565	Italy: Sardinia
*Scanorhynchus forcipatus*	KJ887412	KJ887556	Sweden: Kattegat
*Opisthocystis goettei*	KJ887445	KJ887559	USA: Alabama
*Progyrator mamertinus*	KJ887401	KJ887493	Italy: Sardinia, Carlotto
*Austrorhynchus bruneti*	KJ887405	KJ887498	France: Banyuls-sur-mer
*Rogneda reticulata*	KJ887479	KJ887529	France: Cerbere
*Acrorhynchides robustus*	KJ887458	KJ887517	Germany: Sylt
*Paulodora drepanophora*	KJ887482	KJ887544	South Africa: iSimangaliso NP
Dalytyphloplanida			
*Kytorhynchidae* sp. 1	KC529401	KC529527	Unknown
*Kytorhynchus* sp.	KC529400	KC529526	Unknown
*Coronhelmis multispinosus*	KC529427	KC529555	Unknown
*Cilionema hawaiiensis*	KC529428	KC529556	Unknown
*Parapharyngiella* sp.	KC529405	KC529531	Unknown
*Ptychopera japonica*	MF321751	MF321760	Unknown
*Trigonostomum venenosum*	KC529417	KC529543	Unknown
*Ceratopera gracilis*	KC529422	KC529549	Unknown
*Beklemischeviella angustior*	KC529412	KC529538	Unknown
*Proxenetes quinquespinosus*	KC529406	KC529532	Unknown
*Promesostoma caligulatum*	KC529432	KC529560	Unknown
*Microvahine corallicola*	KC529423	KC529550	Unknown
*Thalassoplanella collaris*	KC529483	KC529614	Unknown
*Brinkmanniella palmata*	KC529424	KC529553	Unknown
*Halammovortex* sp.	KC529437	KC529567	Unknown
***Haloplanella longatuba***	**OP604372**	**OP604371**	**Germany: Sylt**
*Scutariella sinensis*	MF773690	MF773687	China
*Temnosewellia minor*	AY157183	AY157164	Australia
*Castrella truncate*	KC529439	KC529570	Unknown
*Gieysztoria rubra*	KC529480	KC529611	Unknown
*Pseudodalyellia alabamensis*	KC529440	KC529571	Unknown
*Dalyellia viridis*	KC529444	KC529575	Unknown
*Microdalyellia fusca*	KC529453	KC529584	Unknown
*Acrochordonoposthia conica*	KC529487	KC529617	Unknown
*Opistomum arsenii*	KC529491	KC529620	Unknown
*Typhloplana viridata*	KC529484	KC529615	Unknown
*Bryoplana xerophila*	KC529489	KC529619	Unknown
*Phaenocora foliacea*	KC529492	KC529621	Unknown
*Strongylostoma radiatum*	KC529485	KC529616	Unknown
*Adenopharynx mitrabursalis*	KC529520	KC529641	Unknown
*Wahlia macrostylifera*	KC529518	KC529639	Unknown
*Tamanawas kalipis*	MH337259	MH337262	Canada: British Columbia
*Bresslauilla relicta*	KC869832	KC869885	Unknown
*Balgetia semicirculifera*	KC529503	KC529628	Unknown
*Pogaina sinensis*	MK509001	MK509007	Unknown
*Baicalellia canadensis*	KC869833	KC869886	Unknown
*Pterastericola psilastericola*	KC529516	KC529637	Unknown

### Sampling and species identification

A total of 37 samples were taken during two days sampling from intertidal sand in the island of Sylt, Germany. Samples were obtained by digging on the substrate with a 10 cm long shovel. The substrate was kept in zip bags and stored in fridges at 4ºC.

Sampling sites were the beach besides List Harbour (55.015337N, 8.435999E) and the beach in front of Alfred-Wegener-Institute building (55.023745, 8.439049), always during low tide. The collected sediment samples were all coarse sand enriched with variable amounts of organic material. Meiofauna was separated from the sediment using the MgCl_2_ decantation method [18].

**Fig. 2:**
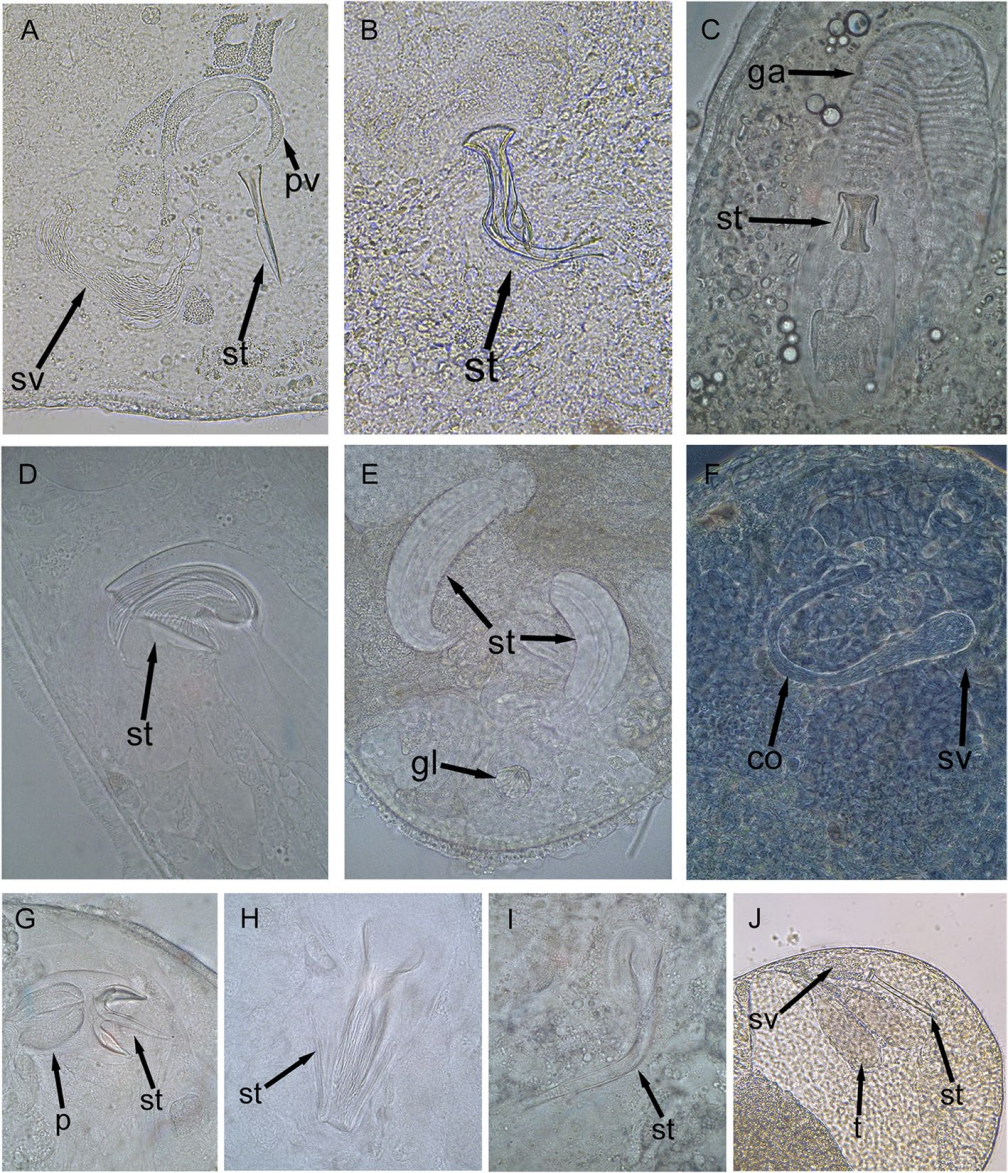
Close−up picture of the copulatory organs, stylets belonging to the identified species from Sylt. Pharynx rosulatus (p), stylet (st), testis (t), vesicula seminaris (sv), prostate vesicle (pv), genital atrium (ga), glandular organ (gl), copulatory organ (co). A. *Marirhynchus longasaeta;* B. *Mariplanella frisia;* C. *Carcharodorhynchus listensis;* D. *Proschizorhynchus gullmarensis;* E. *Psammorhynchus tubulipenis;* F. *Cystiplana paradoxa;* G. *Schizorhynchoides aculeatus;* H. *Schizochilus caecus;* I. *Haloplanella longatuba*. J. *Lonchoplanella axi*

Flatworms were morphologically identified under Leica S APO stereomicroscope and Leica DM 2500 microscope and photographed (stylets) under a portable Leica MC 190 HD attached camera (Fig. 2).

### DNA extraction, amplification and sequencing

For the DNA extraction the DNeasy® Blood & Tissue Kit (QIAGEN) was used. Manufacturer’s instructions were followed, with the exception that DNA was eluted in 60µL of preheated AE elution buffer (60 °C). For samples with low concentration, this protocol was followed by the Amplification of purified genomic DNA protocol from QIAGEN REPLI-g® kit. Thermocycling conditions and primers from Van Steenkiste & Leander [13] were used to sequence markers 18S and 28S (see supplementary material, S1). Sequencing was carried out by Eurofins Genomics (Konstanz, Germany). All new sequences were deposited in GenBank, and sequence accession numbers are provided in Table 1.

### Phylogenetic analyses

Once the sequences were obtained, they were blasted using blastn through ncbi-blast + v.2.12.0 to confirm that platyhelminthes’ DNA was amplified during the PCRs. The rest of the sequences were obtained from GenBank attempting to gather a broad representation of the different families and subfamilies. Those terminals for which both markers (18S and 28S) were available were selected for this study. Several terminals of Proseriata, including *Ciliopharyngiella constricta* were also included to root the tree.

Sequences were visually checked in Geneious v10.2.3 and aligned using MAFFT v.7.305b [19] using the iterative refinement method E-INSI. Single genes (18S, 28S) were concatenated using FASconCAT-G [20, 21]. The maximum likelihood (ML) analysis of the single markers, as well as the concatenated matrix was performed through IQtree v.1.3.11.1 [22, 23], with best fitting models selected by Modelfinder [24] (18S: GTR + F + I + G4; 28S: GTR + F + I + G4). In all analyses, each partition was allowed to have its own set of branch lengths. (-spp option). Support values were estimated based on 1000 bootstrap pseudo replicates (B). iTol v.6. and Adobe Illustrator (2020) were used to edit the phylogenetic trees. The concatenated matrix (18S + 28S) was also analysed through Bayesian inference (BI). For BI analyses, two independent runs of 1,342,000 generations and four chains, each (one cold, three heated) were run in MrBayes 3.2.7 [25]. The most similar models available in MrBayes (-mset option) to those selected by Modelfinder for each partition were applied. All parameters were unlinked, rates were allowed to vary freely over partitions andtrees were sampled every 1000 generations. The runs were stopped when the standard deviation reached the value of 0,007. After discarding 25% first trees as burn-in, trees from the stationary phase were combined to obtain a majority rule consensus and posterior node probabilities [26].

## Supplementary Information

**Additional file 1: S1. **Supplementary table 1

**Additional file 2: SFig. 1. **Phylogenetic relationships between the mayor clades of Platyhelminthes phylum (retrieved from Laumer & Giribet, 2014).

**Additional file 3: SFig. 2. **ML phylogenetic tree inferred from 18S gene. Species provided by this study in red. Bootstrap support values under /beside nodes. Values below 70% not represented.

**Additional file 4: SFig. 3. **ML phylogenetic tree inferred from 28S gene. Species provided by this study in red. Bootstrap support values under /beside nodes. Values below 70% not represented.

**Additional file 5: SFig. 4. **Majority rule consensus tree from BI analysis obtained from the concatenated data set (18S+28S). Posterior probability support values close to each node.

**Additional file 6. **Supplementary figure legends

## Data Availability

The data generated during and/or analyzed during the current study are available in GenBank. Accession numbers are displayed in Table 1.
