# Supplementary material for: New insights into phylogenetic relationships of Rhabdocoela (Platyhelminthes) including members of Mariplanellida
Source: BMC Zool. 2023 Jul 11;8:9. doi: 10.1186/s40850-023-00171-y (PMC10334529; doi:10.1186/s40850-023-00171-y)
Supplement: Supplementary file 1 — Additional file 1: S1. Supplementary table 1 [file 40850_2023_171_MOESM1_ESM.pdf]

Supplementary Table1. Primers used in this study

	**Forward primers (sequence)**	**Reverse primers (sequence)**
18S amplification	TimA (5'-AMCTGGTTGATCCTGCCAG-3')	R18SR (5'-GTTCACCTACGGAAACCTTGTT-3')
18S sequencing	600F (5'-GGTGCCAGCAGCCGCGGT-3')	600R (5'-ACCGCGGCTGCTGGCACC-3')
	1100F (5'-CAGAGGTTCGAAGACGATC-3')	1100R (5'-GATCGTCTTCGAACCTCTG-3')
	18S7F (5'-GCAATAACAGGTCTGTGATGC-3')	18S7FK (5'-GCATCACAGACCTGTTATTGC-3')
28S amplification	LSU5 (5'-TAGGTCGACCCGCTGAAYTTA-3')	LSUD6.3B (5'-GCTGTTCACATGGAACCCTTCTC-3')
28S sequencing	L300F (5'-CAAGTACCGTGAGGGAAAGTTG-3')	L300R (5'-CAACTTTCCCTCACGGTACTTG-3')
	L1200F (5'-CCCGAAAGATGGTGAACTATG-3')	L1200R (5'-GCATAGTTCACCATCTTTCGG-3')

Thermocycling: 95°C for 3m, touch down in 9 cycles (94°C for 30s, 60°C down to 56°C for 30s, 72°C for 1m30s), 31 cycles (94°C for 30s, 55°C for 30s, 72°C for 1m30s), 72°C for 5m
