# Supplementary figures and images for: New insights into phylogenetic relationships of Rhabdocoela (Platyhelminthes) including members of Mariplanellida
Source: BMC Zool. 2023 Jul 11;8:9. doi: 10.1186/s40850-023-00171-y (PMC10334529; doi:10.1186/s40850-023-00171-y)

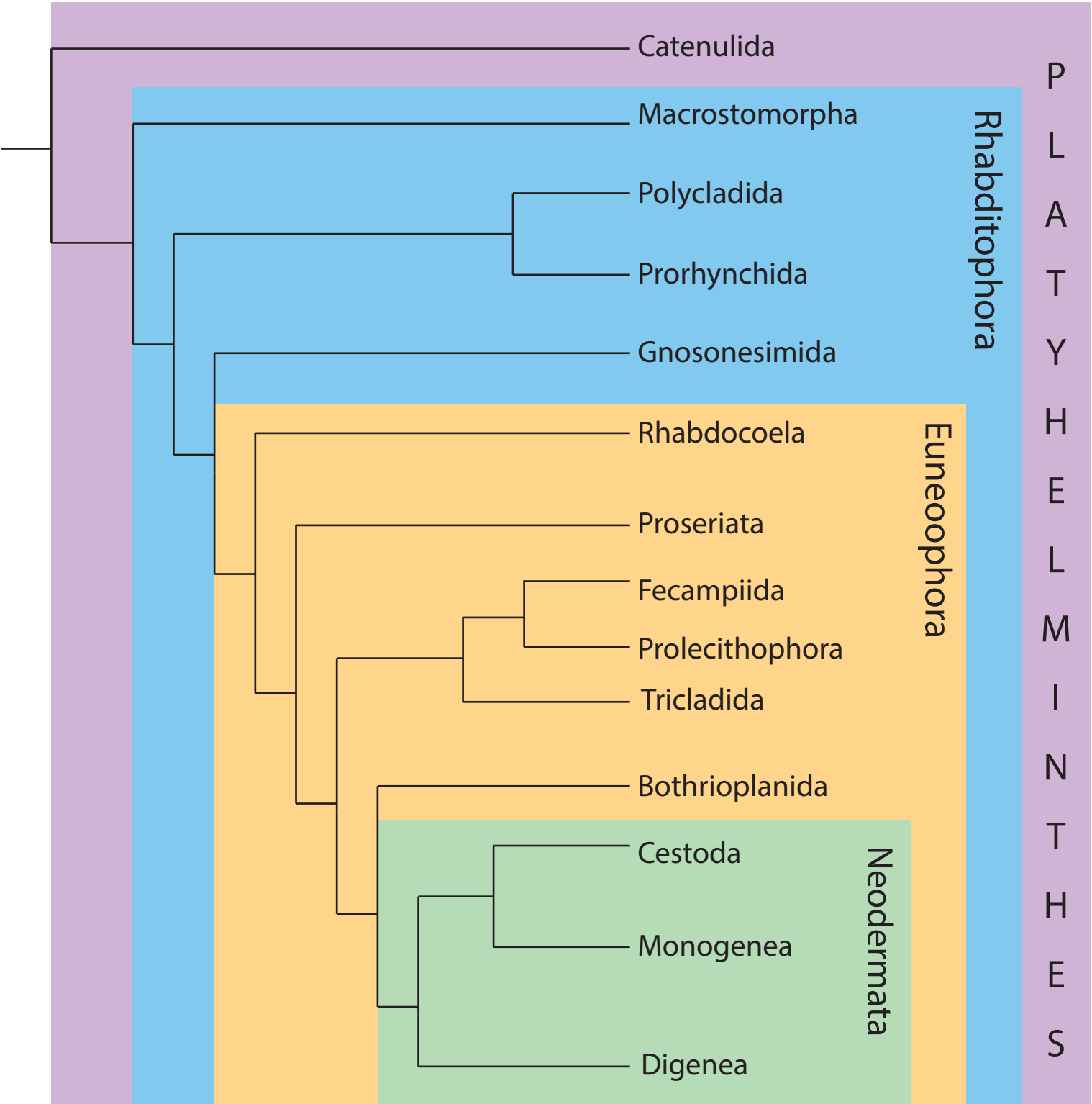

Supplement: Supplementary file 2 — Additional file 2: SFig. 1. Phylogenetic relationships between the mayor clades of Platyhelminthes phylum (retrieved from Laumer & Giribet, 2014). [file 40850_2023_171_MOESM2_ESM.pdf]

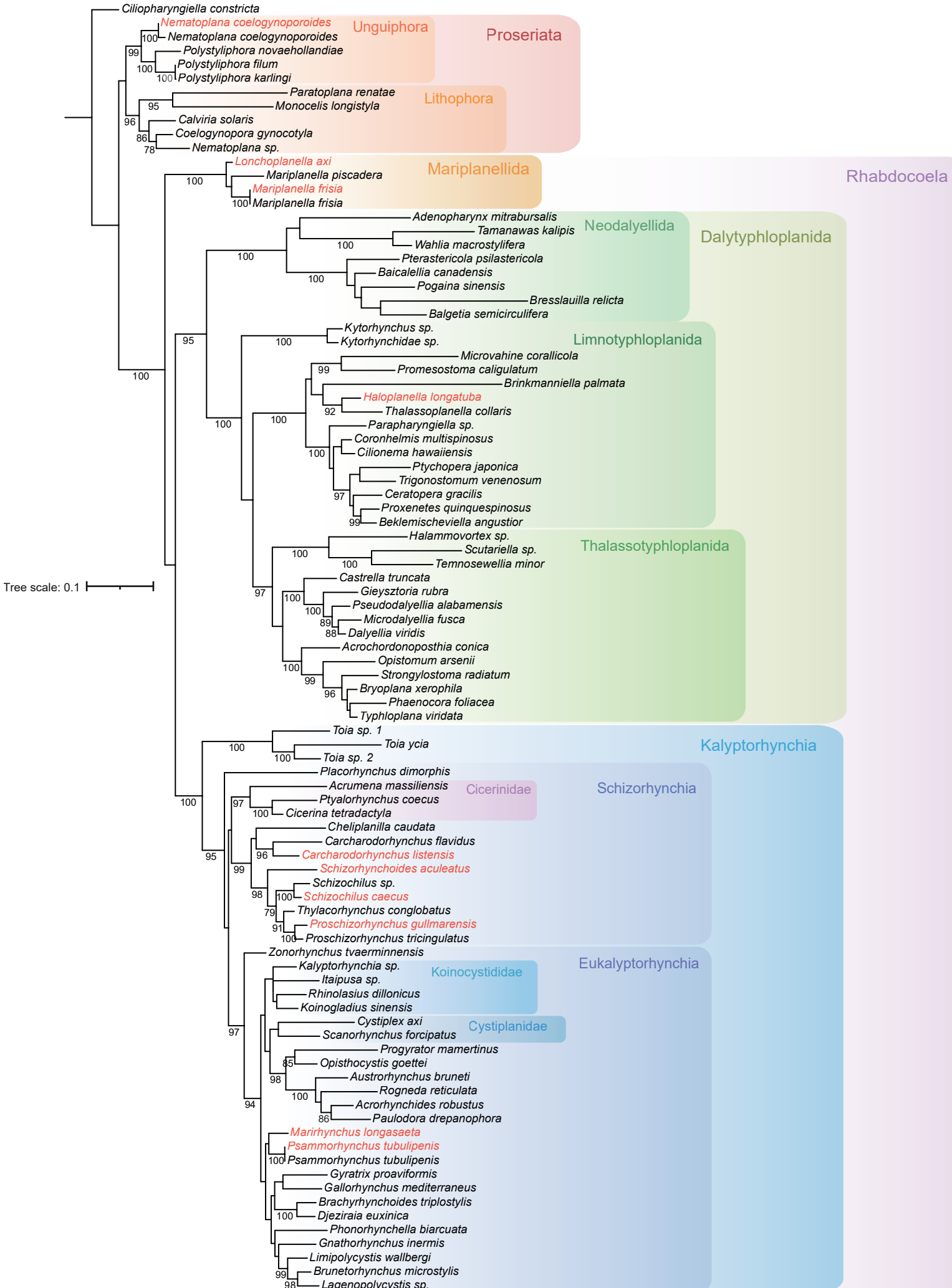

Supplement: Supplementary file 3 — Additional file 3: SFig. 2. ML phylogenetic tree inferred from 18S gene. Species provided by this study in red. Bootstrap support values under /beside nodes. Values below 70% not represented. [file 40850_2023_171_MOESM3_ESM.pdf]

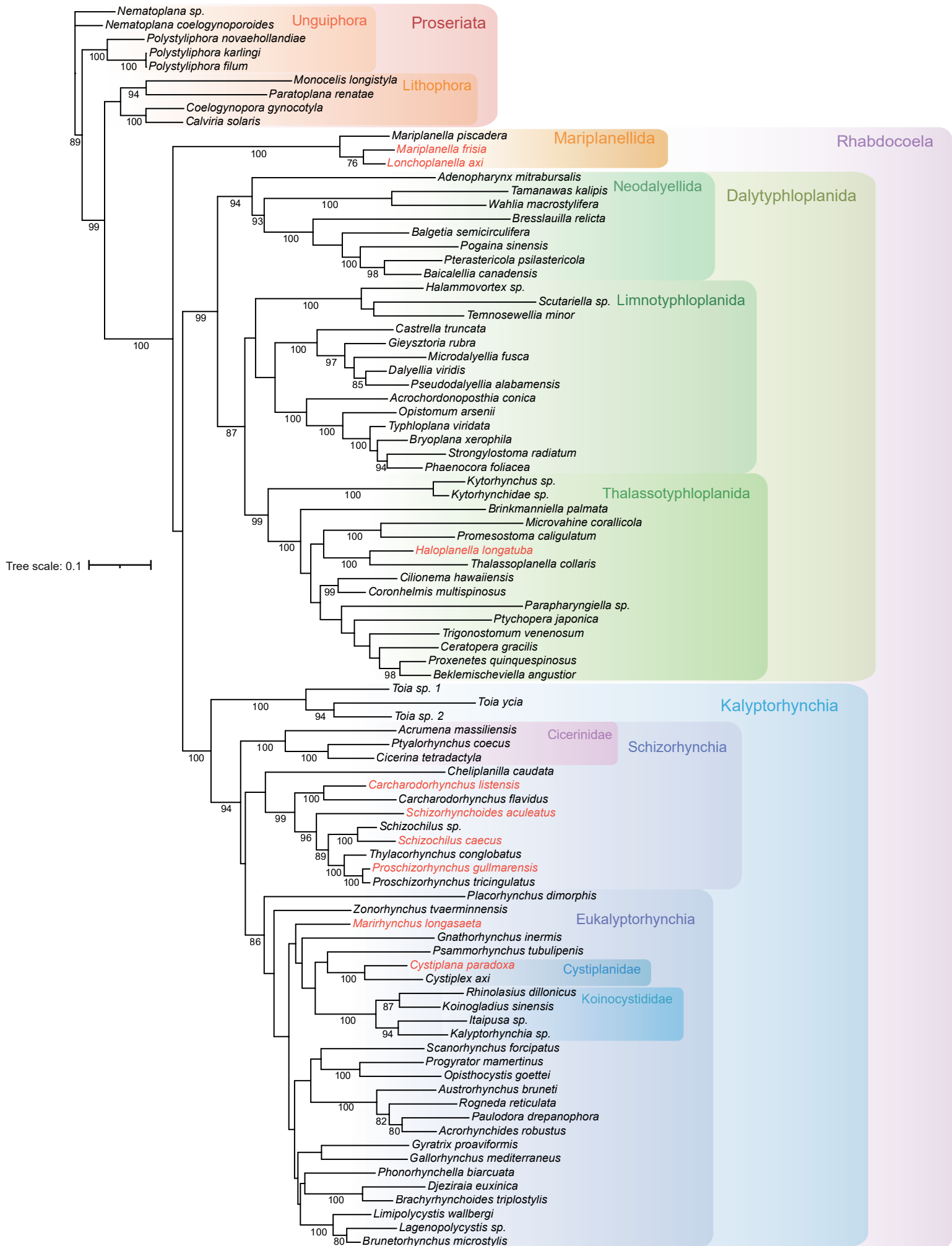

Supplement: Supplementary file 4 — Additional file 4: SFig. 3. ML phylogenetic tree inferred from 28S gene. Species provided by this study in red. Bootstrap support values under /beside nodes. Values below 70% not represented. [file 40850_2023_171_MOESM4_ESM.pdf]

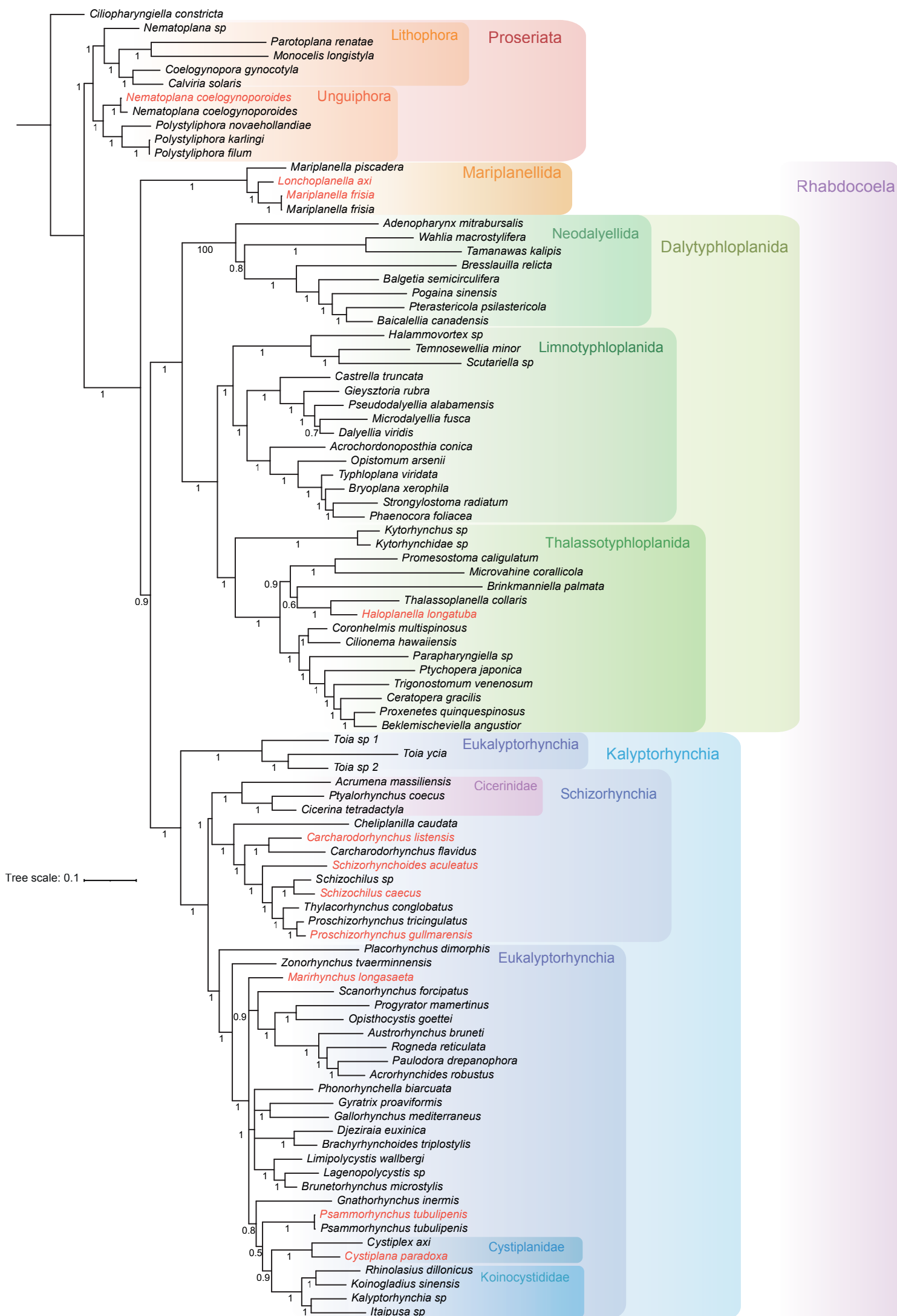

Supplement: Supplementary file 5 — Additional file 5: SFig. 4. Majority rule consensus tree from BI analysis obtained from the concatenated data set (18S+28S). Posterior probability support values close to each node. [file 40850_2023_171_MOESM5_ESM.pdf]
