# Supplementary material for: New insights into phylogenetic relationships of Rhabdocoela (Platyhelminthes) including members of Mariplanellida
Source: BMC Zool. 2023 Jul 11;8:9. doi: 10.1186/s40850-023-00171-y (PMC10334529; doi:10.1186/s40850-023-00171-y)
Supplement: Supplementary file 6 — Additional file 6. Supplementary figure legends [file 40850_2023_171_MOESM6_ESM.docx]

**^Supplementary Figures Legends^**

**^SFig. 1.^** ^Phylogenetic relationships between the mayor clades of Platyhelminthes phylum (retrieved from Laumer & Giribet, 2014).^

**^SFig. 2.^** ^ML phylogenetic tree inferred from 18S gene. Species provided by this study in red. Bootstrap support values under /beside nodes. Values below 70% not represented.^

**^SFig. 3^** ^ML phylogenetic tree inferred from 28S gene. Species provided by this study in red. Bootstrap support values under /beside nodes. Values below 70% not represented.^

**^SFig. 4.^** ^Majority rule consensus tree from BI analysis obtained from the concatenated data set (18S+28S). Posterior probability support values close to each node.^
